# Supplementary material for: Identification of photoperiod-induced specific miRNAs in the adrenal glands of Sunite sheep (Ovis aries)
Source: Front Vet Sci. 2022 Jul 22;9:888207. doi: 10.3389/fvets.2022.888207 (PMC9354845; doi:10.3389/fvets.2022.888207)
Supplement: Supplementary file 1 [file Data_Sheet_1.ZIP › Supplementary file/miRDeep2 file.zip!/miRDeep2 file/Novel_Structure/SP42A1/Novel_658.pdf]

Provisional ID	:	NC_019484.2_26984
Score total	:	1.9
Score for star read(s)	:	-1.3
Score for read counts	:	0
Score for mfe	:	1.6
Score for randfold	:	1.6
Score for cons. seed	:	
Total read count	:	576
Mature read count	:	576
Loop read count	:	0
Star read count	:	0

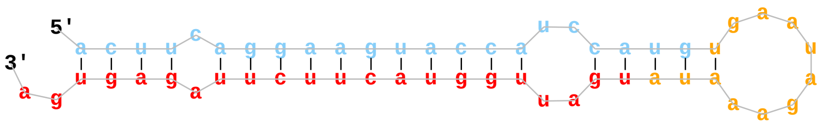

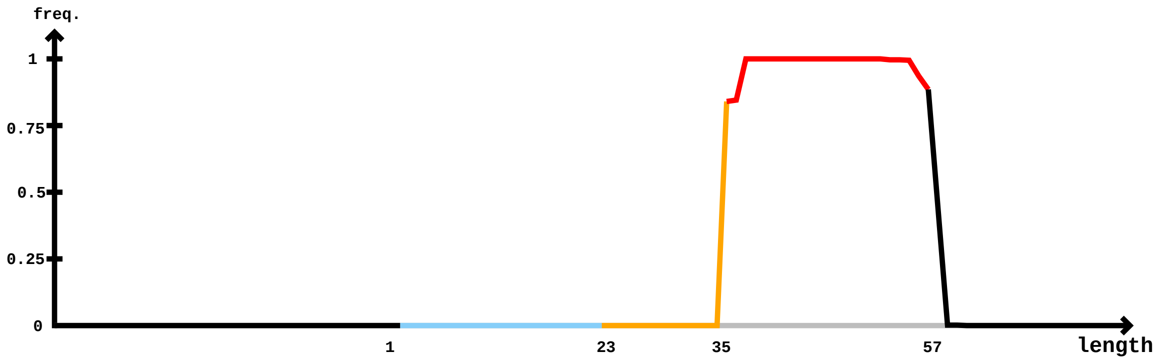


```
                                                   Star                                     Mature
5'- ucaccacauuucacuaugccgcgugcaguauugcacuucaggaaguaccauccaugugaauagaaauaugauugguacuucuuagagugaaguaaugcauagcaaauaca   -3'    exp
    ................(((...(((((.((((.(((((.(((((((((((..(((((........)))))..))))))))))).))))).)))).))))).)))......     reads      mm         sample
    ....................................................................ugauugguacuucuuag.........................     2          0          seq
    ....................................................................ugauugguacuucuuagag.......................     1          0          seq
    ....................................................................ugauugguacuucuuagagu......................     34         0          seq
    ....................................................................ugauugguacuucuuagagug.....................     29         0          seq
    ....................................................................ugauugguacuucuuagaguga....................     373        0          seq
    ....................................................................ugauugguacuucuuagagugaa...................     45         0          seq
    .....................................................................gauugguacuucuuagaguga....................     2          0          seq
    .....................................................................gauugguacuucuuagagugaa...................     1          0          seq
    ......................................................................auugguacuucuuagaguga....................     51         0          seq
    ......................................................................auugguacuucuuagagugaa...................     37         0          seq
    ......................................................................auugguacuucuuagagugaagu.................     1          0          seq
```
